# Hierarchical Optimization Segmentation and Parameter Extraction of Street Trees Based on Topology Checking and Boundary Analysis from LiDAR Point Clouds

**DOI:** 10.3390/s25010188

**Published:** 2025-01-01

**Authors:** Yuan Kou, Xianjun Gao, Yue Zhang, Tianqing Liu, Guanxing An, Fen Ye, Yongyu Tian, Yuhan Chen

**Affiliations:** 1 The First Surveying and Mapping Institute of Hunan Province, Changsha 410114, China; kouyuan@xcyy.net.cn (Y.K.); liutianqing@xcyy.net.cn (T.L.); anguanxing@xcyy.net.cn (G.A.); yefen@xcyy.net.cn (F.Y.); tianyongyu@xcyy.net.cn (Y.T.); 2 Hunan Engineering Research Center of 3D Real Scene Construction and Application Technology, Changsha 410114, China; 3 School of Geosciences, Yangtze University, Wuhan 430100, China; 201900459@yangtzeu.edu.cn (Y.Z.); 202001342@yangtzeu.edu.cn (Y.C.); 4 China Railway Design Corporation, Tianjin 300251, China; 5 Key Laboratory of Mine Environmental Monitoring and Improving around Poyang Lake of Ministry of Natural Resources, East China University of Technology, Nanchang 330013, China

**Keywords:** LiDAR point clouds, street tree segmentation, parameter extraction, topology checking, boundary analysis

## Abstract

Roadside tree segmentation and parameter extraction play an essential role in completing the virtual simulation of road scenes. Point cloud data of roadside trees collected by LiDAR provide important data support for achieving assisted autonomous driving. Due to the interference from trees and other ground objects in street scenes caused by mobile laser scanning, there may be a small number of missing points in the roadside tree point cloud, which makes it familiar for under-segmentation and over-segmentation phenomena to occur in the roadside tree segmentation process. In addition, existing methods have difficulties in meeting measurement requirements for segmentation accuracy in the individual tree segmentation process. In response to the above issues, this paper proposes a roadside tree segmentation algorithm, which first completes the scene pre-segmentation through unsupervised clustering. Then, the over-segmentation and under-segmentation situations that occur during the segmentation process are processed and optimized through projection topology checking and tree adaptive voxel bound analysis. Finally, the overall high-precision segmentation of roadside trees is completed, and relevant parameters such as tree height, diameter at breast height, and crown area are extracted. At the same time, the proposed method was tested using roadside tree scenes. The experimental results show that our methods can effectively recognize all trees in the scene, with an average individual tree segmentation accuracy of 99.07%, and parameter extraction accuracy greater than 90%.

## 1. Introduction

In recent years, three-dimensional (3D) mobile laser scanning (MLS) technology has emerged, providing important support for the construction of tress inventories, real 3D cities modeling [1,2]. With the rapid development and updating of digital twin urbanization, models of major scenes, such as buildings, have been established [3,4,5]. Further establishing a realistic 3D model of detailed road scenes will make the city model more refined, which will play an important role in the development of applications such as detailed urban management and intelligent transportation [6,7]. The construction of tree scenes on both sides of the street essentially requires the extraction of tree parameter information. LiDAR 3D point cloud data provide a reliable data source for fine tree parameter extraction [8]. Therefore, studying tree segmentation and parameters extraction based on LiDAR data has important research value and significance.

The three-dimensional (3D) mobile laser scanning (MLS) technology is a new non-contact, high-precision, and high-density technology that is able to obtain approximate surface real models quickly and actively [9,10,11]. Using profiling scanning techniques and detection lase pulse intensity reflected from the target surface, MLS systems are applied to urban road networks and objects on both roadsides using multiple sensors onboard moving vehicles. There are an increasing number of applications based on MLS point clouds to construct fine models along the road. For example, MLS data are used to extract buildings, road surfaces, trees, traffic signs, poles, and cars [12,13,14]. With the development of cities, many trees are planted along the urban streets. The real-time measurement and management of trees are required. MLS point clouds provide intensive sampling density and sufficient information to recognize the information of trees.

In the street scene, the tree extraction from point clouds is based on rule-based and machine-learning methods [15,16]. Machine learning methods are usually used to classify tree point clouds. Rule-based methods are proposed by designing some special rules to extract tree crowns and trunks to further measure their detailed parameters [17,18]. There are many point cloud segmentation methods, from simple clustering methods to more advanced machine learning techniques using unsupervised and supervised methods. Some methods include K-means, DBSCAN [19], graph-based methods [20], and machine learning [21]. Wu et al. [22] proposed a Voxel-based Marked Neighborhood Searching (VMNS) method for efficiently identifying street trees and deriving their morphological parameters from MLS point cloud data. The method contains six parts, including voxelization, calculating values of voxels, searching and marking neighborhoods, extracting potential trees, deriving morphological parameters, and eliminating pole-like objects. Luo et al. [23] developed a top–down approach to extract individual trees from urban MLS point clouds. It segments tree points using semantic segmentation deep networks and divides the tree points into different sets using clustering. Then, a pointwise direction strategy is embedded to predict the direction vectors to detect the tree center and boundaries to improve the individual trees. Li et al. [24] explored an individual street tree segmentation method by tree nonphotosynthetic components clustering. Gradually removing ground, building objects, and artificial poles, the point clouds of street trees are retained. Then, by clustering nonphotosynthetic components, the main branch can be separated to achieve the complete segmentation of individual trees. Over-segmentation and under-segmentation between trees are common problems in individual tree segmentation. Chen et al. [25] proposed a bottom–up framework for individual tree detection and segmentation. Based on density-based spatial clustering of applications with noise (DBSCAN), the trunks can be detected. Then, the K-Nearest Neighbor (KNN) algorithm and Random Sample Consensus (RANSAC) are combined to correct the trunk detection. The center points of the truck are used as the seed points to obtain the individual trees. In sum, individual tree segmentation methods need to design suitable rules to separate.

The application of ground 3D MLS technology in tree parameter extraction and visualization research is an innovation and expansion of ecology and forestry research. By utilizing ground 3D laser-scanning technology and designing relevant algorithms, basic parameters such as tree diameter and crown volume can be accurately, quickly, and efficiently extracted without damaging trees and saving human resources. In order to better obtain forest information, many scholars have researched tree segmentation and parameter extraction based on remote sensing images and point cloud data. Li et al. [26] proposed a top–down point cloud segmentation (PCS) algorithm to segment (or delineate) a single tree. Wang et al. [27] proposed a single-tree segmentation algorithm using the Euclidean distance between treetops as the segmentation condition is proposed. Recently, deep learning methods have been used for tree segmentation. Zhang et al. [28] designed a deeply supervised tree classification network (DSTCN) using a height-intensity dual attention mechanism to deliver improved classification performance. Further, tree structure parameter extraction is also proposed. Suárez et al. [29] described an approach based on aerial photography and airborne LiDAR to estimate individual tree heights in forest stands using a tree canopy model.Chen et al. [30] proposed the tree top position identification method based on the local maximum algorithm of the point cloud and used the watershed algorithm and template-matching algorithm to locate the trees and extract the tree height and crown width. Fan et al. [31] proposed a quantitative structural model based on the modified AdTree method, and the model was reconstructed to extract the tree volume, diameter at breast height (DBH), and tree height. However, these approaches have limitations in automatically distinguishing individual crown sizes, and further work is needed to estimate diameter distribution and volume [32].

To accurately segment roadside trees and extract tree morphological structure parameters, this paper employs 3D MLS technology, uses tree point clouds as data sources, and develops pertinent algorithms. The contributions are listed in the following aspects:

(1) A voxel-based boundary detection optimization segmentation method is proposed to address the issues of under-segmentation and over-segmentation in existing roadside tree segmentation algorithms. This method optimizes the problem of large individual tree segmentation errors caused by the inaccurate selection of local extremum points based on the traditional canopy height model (CHM).

(2) The method of automatically calculating the volume of a tree crown based on a laser point cloud to realize the lossless automatic calculation of an individual tree crown not only saves manpower but also improves the calculation accuracy, which can provide a data reference for the construction of a virtual simulation model for street scenes.

The rest of this paper is arranged as follows. In Section 2, the related works are introduced in detail. In Section 3, the proposed method is illustrated. The experimental results and analysis are given in Section 4. The comparison with other methods is discussed in Section 5. Then, the proposed method applicability is discussed in Section 6. Finally, Section 7 concludes the whole paper.

## 2. Related Research

### 2.1. Individual Tree Segmentation

Individual tree segmentation has become a hot domestic and international research topic in investigating forest parameters in forestry and the virtual simulation of road scenes [33,34]. Therefore, identifying a separate tree from massive point cloud data is essential in mastering forest resource information and virtual simulation. The quality of individual tree segmentation directly affects the accuracy of tree parameters such as tree height, crown diameter, crown height, and diameter at breast height [35]. The existing individual tree segmentation methods mainly have two directions: one direction is the algorithm for individual tree segmentation based on the CHM, whereas the algorithm for individual tree segmentation directly based on point clouds is another direction.

The individual tree segmentation method based on the CHM generates a digital surface model (DSM) using all point cloud data and a digital terrain model (DTM) using ground points [36]. Then, the CHM was combined with a local maximum algorithm to detect tree tops from the point cloud CHM. Finally, image processing algorithms were used to segment the CHM and obtain an individual tree. Although the CHM-based method has good individual tree segmentation results, due to the complex spatial shape of point cloud data and the influence of noise points, using only local maximum values to define the tree top will inevitably result in over-segmentation results. To overcome this problem, Chen et al. proposed a watershed segmentation method based on label control. This algorithm involves traversing the CHM under a variable window size to identify the top of the tree, using the identified tree vertices as markers to avoid over-segmentation issues in the watershed algorithm [37]. Jing et al. proposed a watershed method based on multi-scale label control, which first determines the size of the tree crown, detects local maximum values of the CHM at multiple scales, generates multi-scale segmentation maps, and finally generates the final tree crown map through integration [38]. Zhao et al. proposed a watershed method based on morphological crown control, which uses morphological algorithms to generate smooth CHM. The local maximum based on smooth CHM is considered as a potential tree vertex to avoid over-segmentation problems caused by local maximum recognition errors. Two watershed transformations are used to reconstruct the basin and extract the crown boundary [39]. Liu et al. [35] proposed a tree crown extraction method based on morphological reconstruction, which integrates the detected tree tops at multiple scales to obtain accurate tree top marker points and uses watershed segmentation algorithms to more accurately detect and segment tree crowns.

The individual tree segmentation method based on point cloud information directly utilizes the 3D information of the point cloud and the geometric features of the individual tree for individual tree segmentation. Ayrey et al. [40] proposed the layer-stacking algorithm, which slices the point cloud data of the forest vertically to obtain the individual tree contours within each segmentation layer. Finally, the contours of each layer of trees are merged to obtain the individual tree. Ferraz et al. [41] proposed applying the mean shift method to individual tree segmentation, utilizing the feature parameters of trees to iteratively move feature points and segment point clouds into target categories corresponding to the features of an individual tree. This method has been widely proven to be an effective individual tree segmentation method based on the 3D information of point clouds. Dai et al. [18] applied the mean shift segmentation method to multispectral LiDAR data. They demonstrated through comparative experiments that the mean shift segmentation method has higher accuracy for individual tree segmentation in point cloud data with multispectral information. Due to the significant impact of parameter settings on the individual tree extraction performance of traditional mean shift segmentation methods, Hui et al. proposed an adaptive mean shift method for calculating kernel bandwidth. Good individual tree extraction results can be achieved without complex parameter settings [42]. Ma et al. proposed an individual tree segmentation algorithm that combines region growth and morphology. Firstly, the region growth algorithm is used for coarse segmentation to generate individual tree edges, and then the morphology segmentation algorithm is used for fine segmentation to solve the problem of under-segmentation caused by unreasonable threshold settings in the region growth method [36]. Huo Lanning et al. improved the individual tree segmentation algorithm based on point clouds in the first introduction and five lines. The point cloud data were horizontally layered, clustered based on local maximum values, and merged into point cloud clusters of different layers according to fusion conditions, improving the segmentation effect of lower-layer trees in complex environments [43]. The individual tree segmentation method based on point cloud data is more direct, which can effectively avoid the errors generated by point cloud data in the interpolation calculation of CHM. By fully utilizing the 3D information of point cloud data, individual tree information can be extracted more accurately, and individual tree structure information can be obtained using the individual tree segmentation results. However, the information integrity of trees is limited by the density of point clouds, which in turn affects the effectiveness of extracting trees.

### 2.2. Tree Parameter Extraction

Tree resources are an essential component of forest resources, and they are important for human survival. Tree parameters are effective indicators for measuring tree resources. Therefore, achieving efficient and accurate tree parameter extraction has vital practical significance. As a new measurement technology, the laser lighting method can easily observe the earth. Due to its unique advantages in estimating forest height and spatial structure, light radar technology was applied to research on estimating forest parameters [44,45,46]. This article explores and attempts to extract relevant parameters from trees.

(1) Tree Height

Tree height refers to the length of a tree from its root and stems to the top of its main stem, which is measured in meters and generally accurate to 0.1 m. Tree height is a factor that represents the height of a tree and is also a major measurement factor for fallen and standing trees. In traditional methods, tree height is mainly measured using a height gauge. In this paper, point cloud data are used for extraction. The measurement of the height of a living tree is relatively difficult. When the height of the tree is greater than two meters, it is necessary to use a height gauge for measurement. Due to the difficulty in seeing both the top of the tree and the base of the tree trunk at the same time, measurement errors are invisibly increased. The calculation of tree height based on point cloud data can not only reduce errors but also be convenient. The specific algorithm is as follows: the height difference between the highest point of the tree point cloud and the root node is the tree height [47,48].

LiDAR data can directly extract tree height information, and different data processing methods can affect the final result of tree height recognition. Individual tree overestimation algorithms can be divided into three categories. The first category is to directly use the local maximum algorithm to search crown vertices in the CHM, including fixed and variable window sizes [49,50]. The second type first determines the boundary of the tree crown and then calculates the maximum value within the boundary as the tree height [51]. The third type is to determine the position of individual trees through field surveys and search for the corresponding crown vertices in CHM [52].

(2) Diameter at Breast Height (DBH)

The DBH is an important parameter for investigating forest resources, usually the diameter of the tree trunk at a distance of 1.3 m from the root of the tree trunk. The main extraction process includes searching for point cloud data in the trunk segment at a distance of 1.3 m ± 0.5 m from the tree root, fitting these point cloud data, and the diameter of the fitted circle is the DBH.

If the chest diameter is extracted from a cross-section, the average of the maximum and minimum values in the measurement data is used as the chest diameter. If there is a bifurcation at the extraction point, we select the branch with the largest radius for the calculation of chest diameter. At present, the common methods of DBH extraction are DBH estimation by circle fitting based on the Hough transform [7] and DBH estimation by least square circle fitting [53].

(3) Tree Location

The commonly used individual tree positioning recognition algorithms include local maximum, binarization [54], template matching [55], and scale analysis [34]. Among them, the local maximum method assumes that the center of the crown is higher than the edge of the crown. The local maximum filtering first defines a window of a certain size and then uses this window to search for the canopy image, selecting the maximum value of the local spectrum as the center point of the canopy. The global threshold binarization method attempts to transform grayscale into a black-and-white binary image. By analyzing the grayscale histogram of the image, Dralle et al. selected an appropriate threshold to distinguish the canopy and background and achieved individual tree localization [56]. The template-matching method is based on the shape characteristics of the tree crown, matching the crown shape with the gray distribution of the image. Scale selection is one of the key factors in tree detection. For images with fixed spatial resolution but different crown shapes, it is difficult to detect tree crowns of different crown sizes simultaneously. The segmentation results of each scale can be modified and integrated to achieve individual tree crown extraction, achieving the goal of individual tree localization.

(4) Crown Diameter

The individual tree crown refers to the outer layer of the leaves of an individual tree. At present, the commonly used crown extraction algorithms are mainly divided into two categories as follows: one is to extract the crown of an individual tree through certain growth conditions based on the positioning results of an individual tree, such as the ray method [57], seed area growth algorithm and watershed segmentation algorithm [58]. The other is to draw the crown contour directly from the crown shape according to the characteristics of the image itself, such as the valley searching method [59], multi-scale analysis method, and active contour model method.

## 3. Material and Method

### 3.1. Dataset Acquisition

In order to test the effectiveness of the proposed method, two datasets containing street trees point clouds were used. Dataset 1 is the street point cloud data collected in Changsha City, Hunan Province. Dataset 2 is part of the data from the 7th National LiDAR Conference Data Processing Competition. According to the point cloud data obtained by MLS scanning, the ground point cloud that occupies the vast majority will seriously affect the subsequent single wood segmentation process. Therefore, we use the cloth simulation filter (CSF) algorithm [60] to preprocess the point cloud data to achieve effective filtering of ground point clouds.

**Figure 1:**
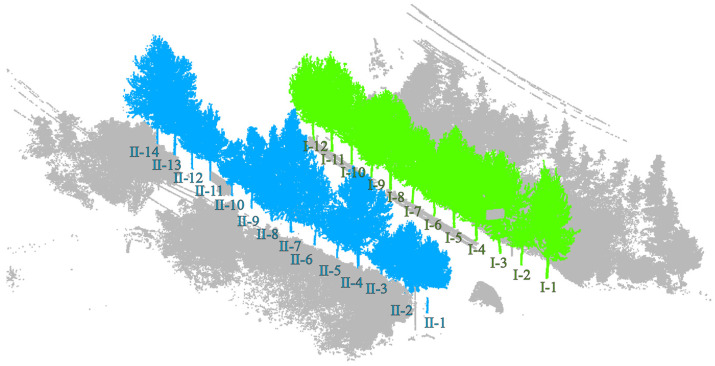
Display of street tree test dataset 1.

Dataset 1: To facilitate the acquisition of point cloud data from street trees, a vehicle-mounted 3D laser scanner is used for point cloud data collection. Due to severe obstruction between trees and between leaves and branches, to obtain more accurate point cloud data of roadside trees, the scanner is placed above the vehicle platform for multi-point scanning to obtain comprehensive point cloud data. The obtained street tree data are shown in Figure 1. Among them, there are 321,332 points in this scene, and there are 26 roadside trees in the scene.

**Figure 2:**
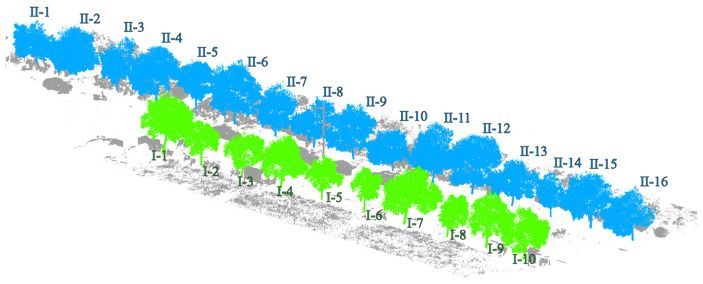
Display of street tree test dataset 2. (Points colored with blue and green are trees. Points colored with gray are background noise.).

Dataset 2: As shown in Figure 2, the data are the laser point cloud of the road scene around the School of Surveying and Mapping and the School of Civil Engineering of Henan Polytechnic University obtained by using the Zhonghaida ARS1000L from Guangzhou, China vehicle-mounted laser scanner on 17 August 2023, and the point cloud includes all the ground feature information obtained by the vehicle-mounted system, such as the ground, street trees, buildings, green belts, basic public facilities, pedestrians, etc., among which the street trees include independent street trees and connected street trees, and a small number of street tree point clouds will be missing due to occlusion and other reasons. The driving speed of the vehicle-mounted LiDAR system is 35 km/h when collecting data. To avoid the sensitivity of the data coordinates, the vehicle-mounted LiDAR point cloud coordinates are converted into the station coordinate system of the ground laser LiDAR. At the same time, in order to ensure the reliability of the assessment benchmark data, the UAV radar system and the Zhonghaida Riegl-VZ1000 from Guangzhou China ground-based 3D laser scanner were used to obtain all-round data in the survey area on the same day.

### 3.2. Overframe of Roadside Tree Segmentation Algorithm

**Figure 3:**
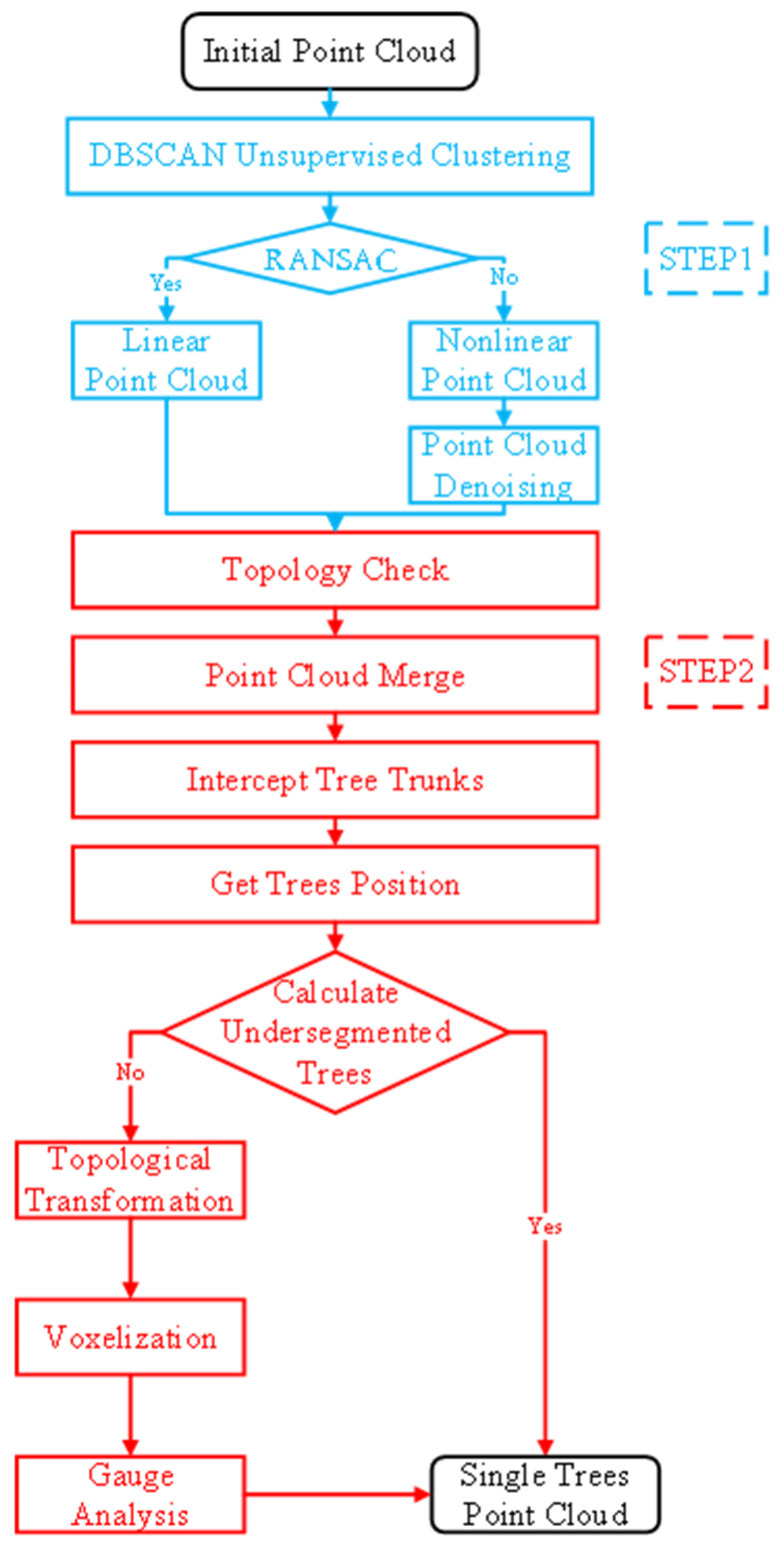
Procession of tree segmentation.

The processing process of roadside tree segmentation is shown in Figure 3. It consists of two steps: pre-segmentation of roadside trees and optimizing inaccurate processing for roadside trees.

### 3.3. Tree Pre-Segmentation

**Figure 4:**
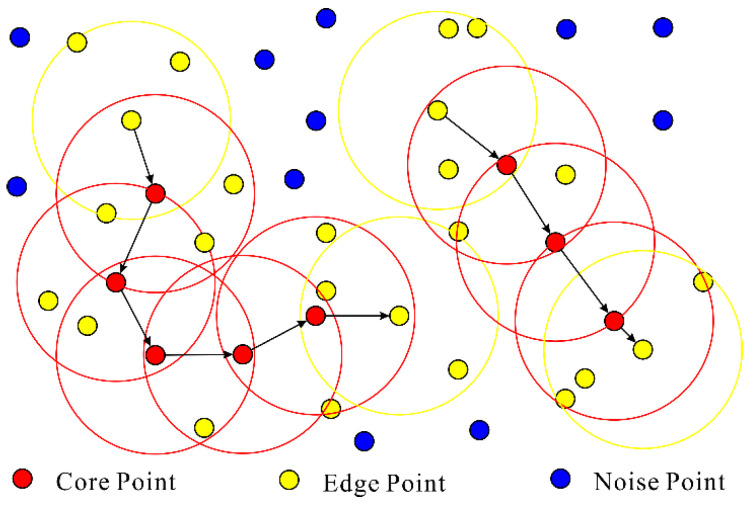
Schematic diagram of the DBSCAN algorithm.

DBSCAN is a density-based spatial clustering algorithm for measurement data. Due to its ability to effectively solve the interference of noisy data, there is no need to set the number of targets in advance, and it enables the discovery of clustering targets with arbitrary convex shapes in dense point cloud data. This algorithm is widely applicable in the clustering process of point cloud data. DBSCAN uses a set of parameters about ‘neighborhood’ to describe the compactness of sample distribution, which defines all point cloud data as core points, boundary points, and noise points, with different types of points corresponding to different point cloud data densities. Interference point cloud data and effective point cloud data can be distinguished by dividing different types of point cloud data through density. The principle of the DBSCAN algorithm is shown in Figure 4.

**Figure 5:**
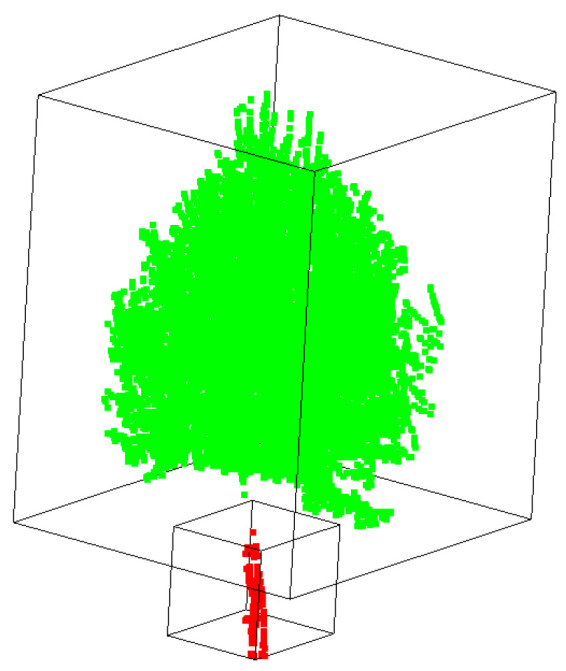
Display of tree segmentation.

Due to severe obstruction between tree branches, some tree trunks are missing, resulting in over-segmentation of the trees, as shown in Figure 5. To avoid the situation where some over-segmentation results are filtered out during the point cloud denoising process, resulting in lower accuracy of the final individual tree segmentation, this article uses the random sample consensus (RANSAC) algorithm to detect the linear state of clustering results. Among them, the distance threshold from the set point to the straight line is 0.4 m. Finally, the average number of points in DBSCAN nonlinear clustering results is statistically analyzed. Then, the clustering results below one third of the average number of points are identified as noise for filtering.

### 3.4. Segmentation Optimization Based on Topology Checking and Boundaries

The pre-segmentation results are inaccurate enough with some over-segmentation or under-segmentation. In this part, we will optimize the initial results to improve the segmentation accuracy. Due to the severe occlusion between tree branches, there are some over-segmented point cloud data in the initial tree segmentation results. To avoid the impact of over-segmentation on the final segmentation accuracy, this article conducts XOY surface projection on the initial segmentation results and topology overlay analysis on the projection results to identify the possible over-segmentation of trees in the initial extraction results. When two or more cluster projection results completely overlap, we merge the completely overlapping point cloud data to optimize the problem of tree point cloud over-segmentation caused by uneven point cloud density caused by branch covering.

The diameter at the breast height (DBH) of a tree refers to the diameter of the trunk at a distance of 1.3 m from the root. Therefore, this article intercepts point cloud data within the interval of [1.25 m, 1.35 m] and uses the least squares circle fitting method to process the intercepted data. By minimizing the sum of squares of the length of each line segment from the actual contour points to the corresponding points on the fitted ideal circle and using the center of the ideal circle as the exact position point data of the tree, precise positioning of the street tree in the scene is achieved.

In practical scenarios, there is a situation where branches and leaves adhere to each other between roadside trees, which still results in under-segmentation where multiple trees are classified as the same tree in the optimized segmentation results. First, whether trees are under-segmented was judged by analyzing the corresponding relationship between tree location and clustering results. Secondly, suppose there are multiple trees in the clustering result. In that case, the rotation matrix is defined by the spatial position information of multiple trees to correct the main direction of the point cloud data in the clustering result. Then, the updated point cloud data are the progressive aspect, and the under-segmented individual tree is searched by detecting the density change between voxels to obtain the critical voxels between the under-segmented individual trees. Finally, the critical voxel center position between under-segmented individual trees is segmented to complete the entire tree under-segmentation optimization process.

**Figure 6:**
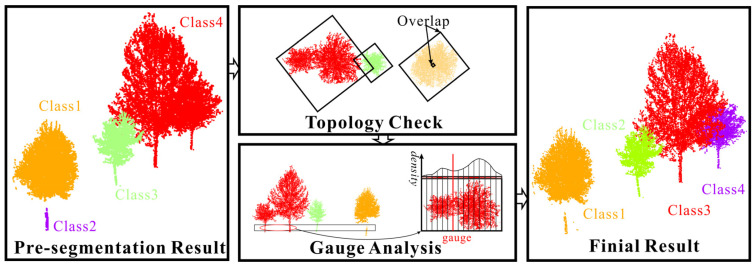
Optimization process diagram.

As shown in Figure 6, the method can effectively optimize the handling of over-segmentation or under-segmentation problems during the pre-segmentation process.

**Figure 7:**
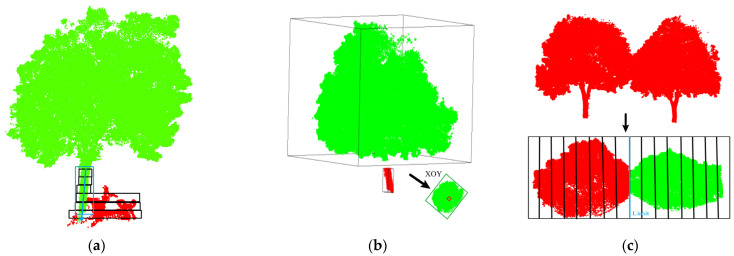
Topology checking and gauge analysis. (**a**) Topology checking of the growth direction of the trunk; (**b**) XOY canopy topology relationship analysis; (**c**) Gauge Analysis.

Specifically, topology checking and gauge analysis are used to solve the problem of over-segmentation and under-segmentation of single trees. The topology checking mainly includes the topology examination of the growth direction of the trunk of a single tree and the topology relationship analysis of the XOY canopy, as shown in Figure 7. The trunk growth direction of a single tree is based on several voxels divided along the Z-axis direction to obtain the trunk growth topology framework, and the topology skeleton is used to achieve precise segmentation of the trunk to remove the interference of ground objects around the trunk, as shown in Figure 7a. Due to the lack of point clouds that may occur during the scanning process of point cloud data, the phenomenon of single wood over-segmentation may occur in the initial segmentation process. The topology relationship of the XOY canopy was combined by analyzing the topology relationship of the XOY projection surface of the initial segmentation results, as shown in Figure 7b. The limit analysis is mainly used to deal with the point cloud of under-segmented trees, which is divided into several voxels along the trunk connection direction based on the XOY plane, and the boundary position between trees is obtained by analyzing the density change in point clouds between voxels so as to achieve an accurate segmentation of under-segmented trees, as shown in Figure 7c.

### 3.5. Tree Parameter Extraction Algorithm

Individual tree parameter extraction based on point cloud data mainly includes three parameters: tree height, DBH, and crown area. Under the premise of accurate individual tree segmentation, the tree height is usually the difference between the highest and lowest points of the point cloud data of an individual tree. Assuming that there are *N* trees in the study area, the point cloud data in the *i*th tree can form a set *P_i_*; then, the height of the *i*th tree is *H_i_*, and the height calculation formula can be expressed as

(1) $$H_{i}=\max\left(P_{i}\right)-\min\left(P_{i}\right)$$

After obtaining the height of each tree in the study area, the arithmetic average height can be calculated to obtain the average height of the stand *AvgH*. The calculation formula can be expressed as

(2) $$AvgH=\frac{\sum_{i=1}^{N}H_{i}}{N}$$

**Figure 8:**
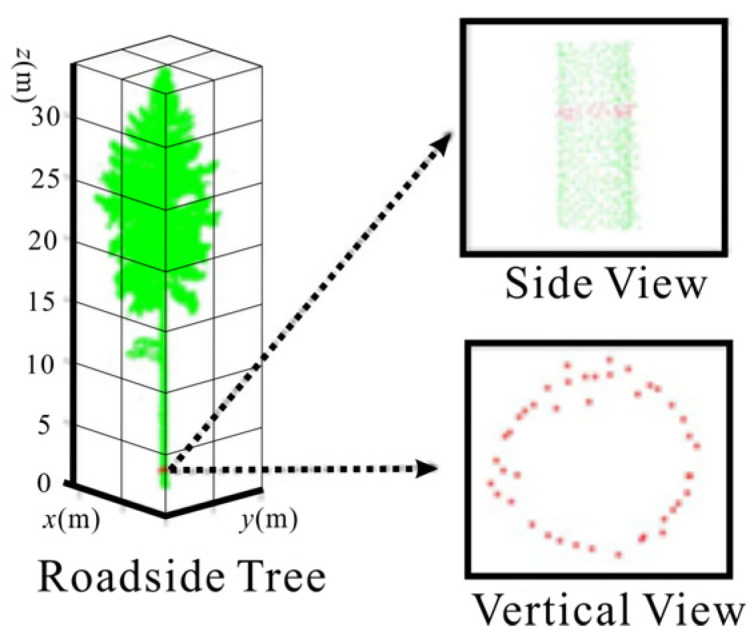
Analysis results of chest diameter point cloud data.

The DBH refers to the diameter of the trunk at a distance of 1.3 m from the root of the tree. Usually, point cloud data within the range of [1.25 m, 1.35 m] are taken as the initial point cloud data for estimating the diameter at breast height, as shown in Figure 8. Therefore, we choose the least squares circle fitting method to obtain the final fitted DBH by minimizing the sum of the squares of the length of each line segment from the actual contour points to the corresponding points on the fitted ideal circle.

Assume the coordinates of the center of the circle are (*a*, *b*), and the radius of the circle is *r*. Therefore, the square of the distance from the point to the edge of the circle is the sum of the difference between two squares of the radius *δ*^2^:

(3) $$\sum \delta^{2}=\sum {\left[X_{i}^{2}+Y_{i}^{2}+AX+BY+C\right]}^{2}$$

Among them, *A* = −2*a*, *B* = −2*b*, *C* = *a*^2^ + *b*^2^ − *r*^2^.

**Figure 9:**
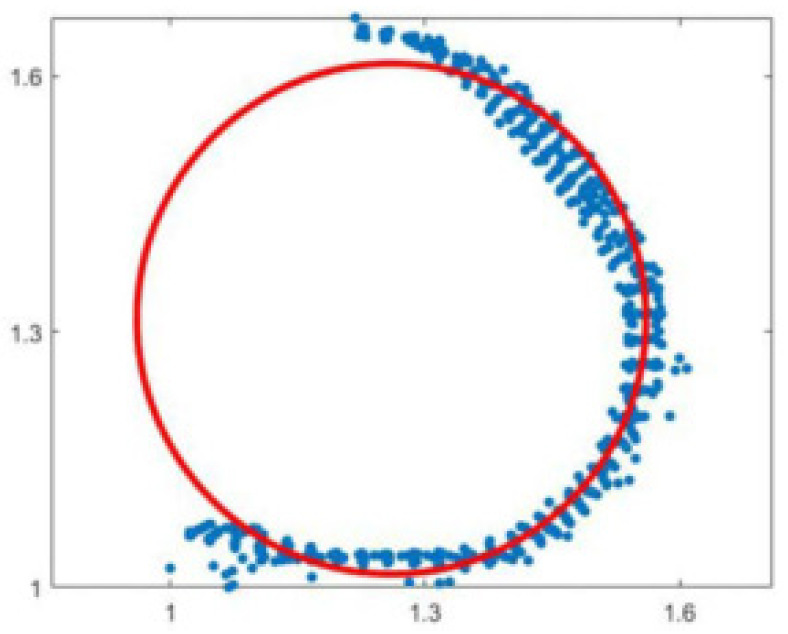
Effect of least squares circle fitting method.

At this point, only parameters *A*, *B*, and *C* are required to be made for $\sum \delta^{2}$ when the value of *θ* is the smallest, and the effect of fitting a circle is the best. Immediately, $\sum \delta^{2}$ is used to calculate the partial derivatives of *A*, *B*, and *C*, respectively, and make the partial derivatives equal to zero to obtain the extreme points to minimize the sum of the difference between the two squares. The fitting circle effect is better when the input point cloud data match the chest diameter position well, as shown in Figure 9.

**Figure 10:**
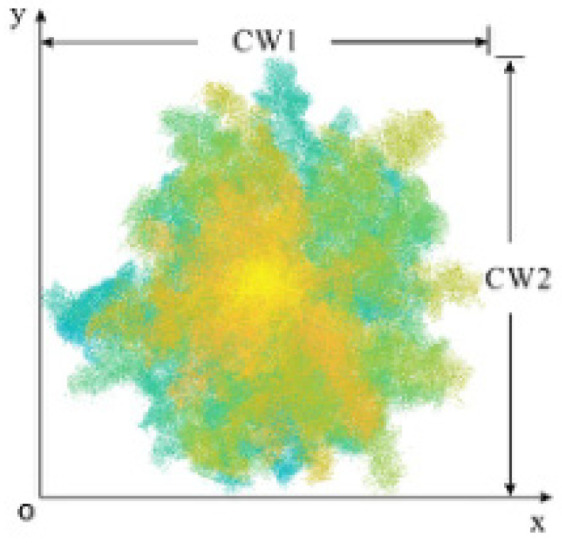
Schematic diagram of crown diameter detection.

The crown, as the main component of a tree, is an essential indicator for measuring its growth. The parameter values are usually represented by the crown width, which generally refers to the average width of the tree crown in the east–west and north–south directions, as shown in Figure 10. However, considering that the obtained tree crown point cloud may be incomplete or irregular, this paper projects the individual tree point cloud data onto a two-dimensional (2D) plane. Then, it calculates the 2D convex polygon of the individual tree from the discrete point data of the 2D plane. The convex points on the 2D convex polygon are connected, and the spacing between the convex points is calculated in turn. Finally, the individual tree crown can be obtained by screening the maximum spacing between the convex points. In this paper, the minimum convex polygon is obtained by constructing the plane discrete point cloud data set using the fast package method.

**Figure 11:**
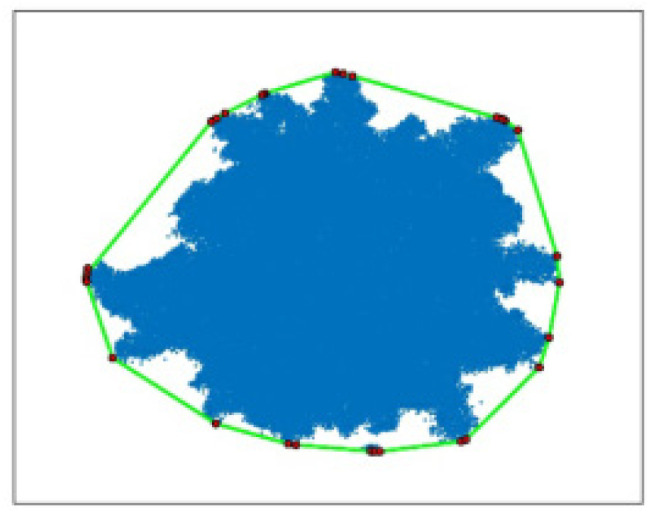
Schematic diagram of crown convex hull method.

As shown in Figure 11, the crown diameter of the final individual tree based on the crown convex hull method is used. For the body, first search for and obtain the two diagonal points of the convex hull based on the principle of prioritizing the X coordinate. Then, the two diagonal points are connected, and the point cloud set is divided into two sub-point clouds. The point farthest from the diagonal convex hull line from the sub-set is selected as the new convex hull point. Iterating the above process until no new convex hull points appear, obtain the set of convex hull vertices in the 2D plane. Finally, the paired convex hull points with the maximum spacing value are selected and calculated to obtain the crown area of an individual tree. The calculation formula can be expressed as

(4) $$P=\max\left(\sqrt{{\left(x_{j}-x_{i}\right)}^{2}+{\left(y_{j}-y_{i}\right)}^{2}}\right)$$

where $\left(x_{i},y_{i}\right)$ and $\left(x_{j},y_{j}\right)$ are any two points in the convex hull vertex, $\left(i,j=1,2,\cdots ,n\right)$. *P* is the crown diameter of the final individual tree.

## 4. Experiment Analysis

### 4.1. Evaluating Indicator

In the individual tree segmentation experiment, the results of individual tree segmentation are mainly quantitatively analyzed through tree-oriented and point-oriented methods. Both methods were quantitatively analyzed using *Precision* (*P*), *Recall* (*R*), and *F*_1_ methods. *P* represents the proportion of extracted values to the actual value, while *R* represents the proportion of extracted values to the extracted actual value. The calculation formula is as follows:

(5) $$P=\frac{tp}{tp+fp}$$

(6) $$R=\frac{tp}{tp+tn}$$

(7) $$F_{1}=\frac{2*P*R}{P+R}$$

(8) $$AI\mathrm{oU}=\frac{A\cap B}{A\cup B}$$

Among them, when facing point objects, *tp*, *tn*, and *fp* represent true positive points, true negative points, and false positive points, respectively. *AI*oU represents the intersection rate between *A* and *B*, where *A* and *B* represent the crown area from the test point cloud and the crown area from the tree point cloud by manual measuring, respectively. When facing tree objects, when more than 80% of the points of the tree are segmented, it is considered that the tree is correctly segmented as *tp*. When less than 50% of the points on the tree are segmented, it is considered that the tree has not been correctly segmented as *fp* and vice versa as *tn*.

### 4.2. Experimental Result of Dataset 1

**Figure 12:**
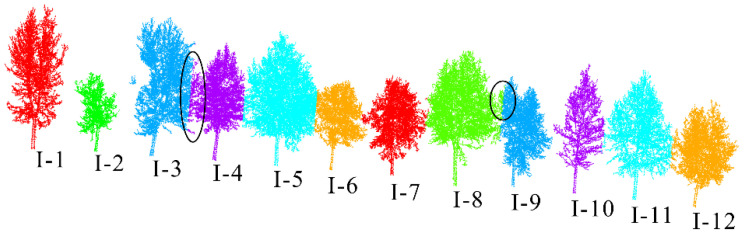
Effect of individual tree segmentation on the left side of the road in dataset 1.

**Figure 13:**
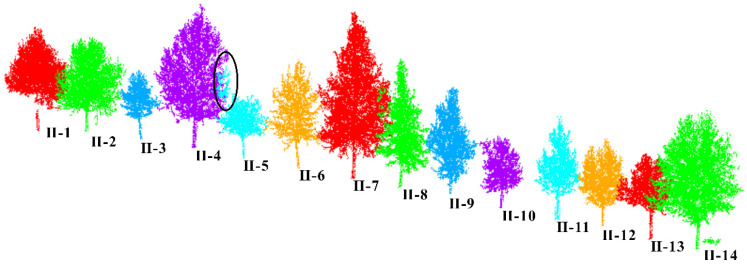
Effect of individual tree segmentation on the right side of the road in dataset 1.

This article uses the proposed individual tree segmentation algorithm to process the dataset and verify its effectiveness. The quantitative evaluation results are shown in Table 1, and the segmentation effects of individual trees on both sides of the road are shown in Figure 12 and Figure 13, respectively.

The test results indicate that the proposed algorithm can accurately extract all street trees in the test scenario with an average extraction accuracy of 99.07%. In addition, there are a few instances of the tree branch and leaf point cloud segmentation in the qualitative results, but the segmentation of point cloud parts has a small impact on the overall segmentation accuracy of the tree. Specifically, in Figure 9, due to the occurrence of edge branches and leaves being divided into I-4 in I-3, the *R*-value of I-3 is relatively low. At the same time, there is also a situation where the fine division of tree boundaries is not high (I-8 and I-9 in Figure 9; II-4 and II-5 in Figure 10), resulting in some branch and leaf point clouds of I-9 and II-4 being divided into I-8 and II-5, respectively. However, due to the difference in the number of point clouds between I-8, I-9, and II-4, II-5, the misclassification of point clouds has a significant impact on the accuracy of II-5 small trees, and the impact on I-8 and II-4 is not significant in quantitative evaluation.

**Figure 14:**
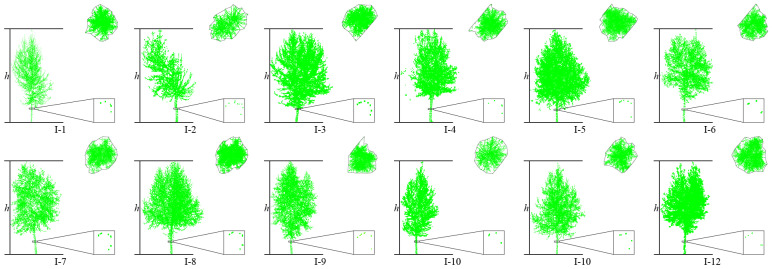
Tree parameter extraction results on the right side of the road in dataset 1.

**Figure 15:**
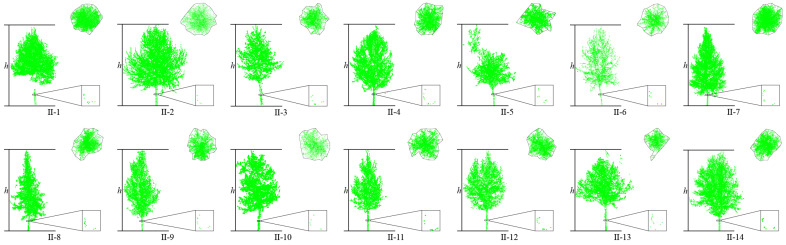
Tree parameter extraction results on the left side of the road in dataset 1.

In addition, this article uses the parameter extraction method introduced in Section 3.3 to extract the tree height, tree diameter at breast height, tree radius, and tree crown area from the individual tree segmentation results. Among them, because the point cloud density of the trunk and center of the tree is large, while the point cloud density of the surrounding branches and leaves is small, the under-segmentation phenomenon in the segmentation results mostly occurs in the branch and leaf part, which makes the branch and leaf under-segmentation phenomenon in the qualitative results of I3, I9, and II4 not obvious in the quantitative results. The parameter extraction results are shown in Table 2, and the parameter results of the trees on both sides of the road are shown in Figure 14 and Figure 15, respectively.

### 4.3. Experimental Result of Dataset 2

**Figure 16:**
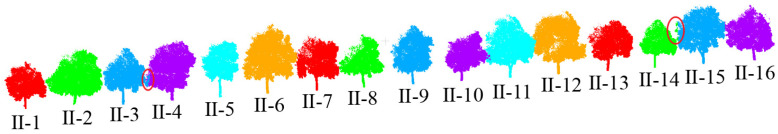
Effect of individual tree segmentation on the right side of the road in dataset 2.

**Figure 17:**
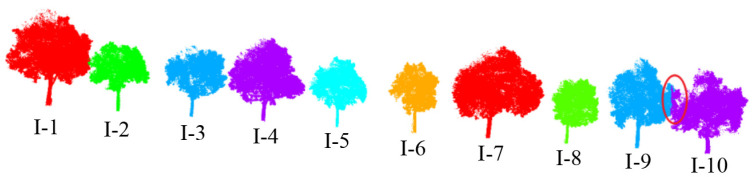
Effect of individual tree segmentation on the left side of the road in dataset 2.

This article uses the proposed individual tree segmentation algorithm to process the dataset and verify its effectiveness. The quantitative evaluation results are shown in Table 3, and the segmentation effects of individual trees on both sides of the road are shown in Figure 16 and Figure 17, respectively.

**Figure 18:**
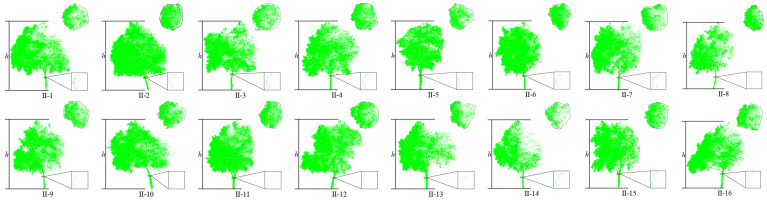
Tree parameter extraction results on the right side of the road in dataset 2.

**Figure 19:**
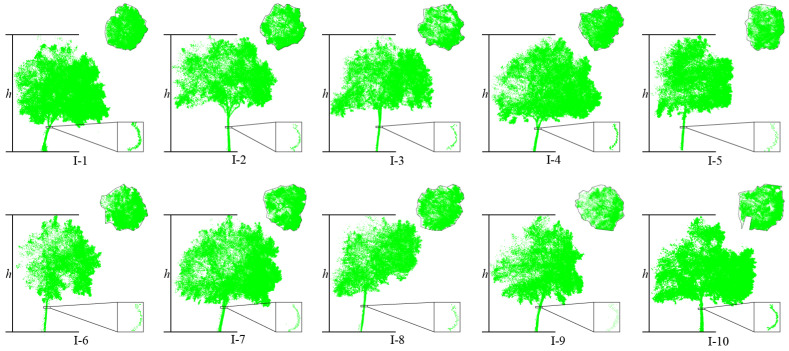
Tree parameter extraction results on the left side of the road in dataset 2.

As shown in Figure 18 and Figure 19 and Table 3, the test results show that the proposed algorithm can accurately extract all street trees in the test scene, and the average extraction accuracy is 99.66%. In addition, there was a small number of misdivisions in the qualitative results. Specifically, in Figure 14, the R value is relatively low because the II-15 part of the branches and leaves is classified as II-14. At the same time, I-9 and I-10 have blurred tree boundaries. In general, the proposed algorithm can effectively process the street tree point cloud, and the relevant parameter extraction method is used to effectively extract the tree height parameters.

In addition, as shown in Table 4, the accuracy of the parameter extraction results for all trees was above 90%. The reason for this is that there is a small amount of ambiguity in the boundary of the division of some crown areas

## 5. Comparison Analysis

**Figure 20:**
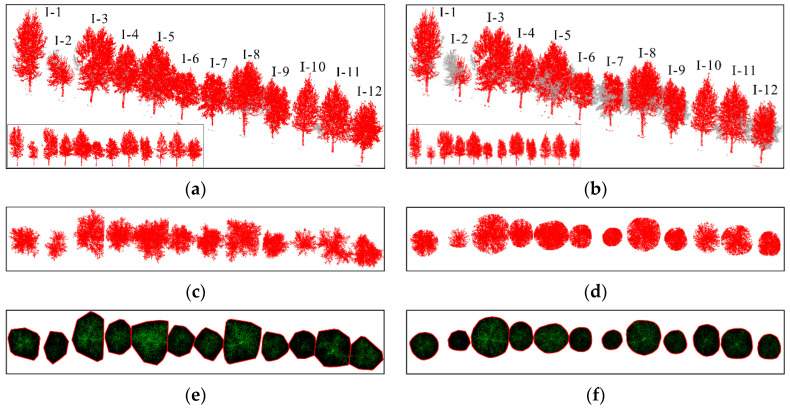
Comparison of individual tree segmentation effects on the left side of the road. (**a**) The segmentation effect of the individual tree algorithm of our method; (**b**) Comparison algorithm results of the individual tree segmentation; (**c**) Top view segmentation results of our method; (**d**) Top view results of comparison algorithm; (**e**) The convex hull of the tree crown results of our method; (**f**) The crown convex hull results of Comparison algorithm.

**Figure 21:**
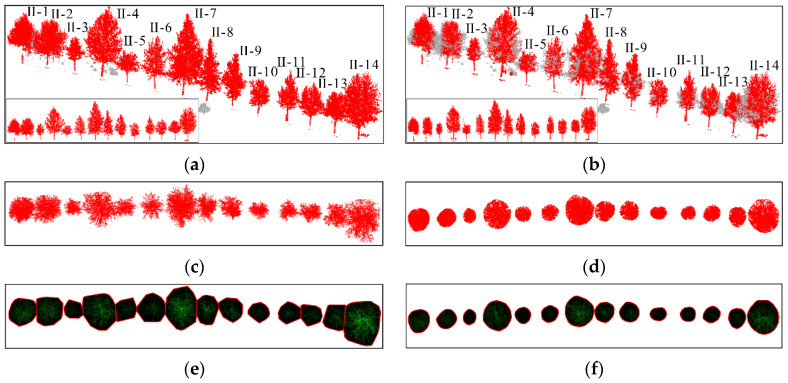
Comparison of individual tree segmentation effects on the right side of the road. (**a**) The segmentation effect of the individual tree algorithm of our method; (**b**) Comparison algorithm results for individual tree segmentation effect; (**c**) Top view segmentation results of our method; (**d**) Top view results of comparison algorithm; (**e**) The convex hull of the tree crown results of our method; (**f**) The crown convex hull results of Comparison algorithm.

**Figure 22:**
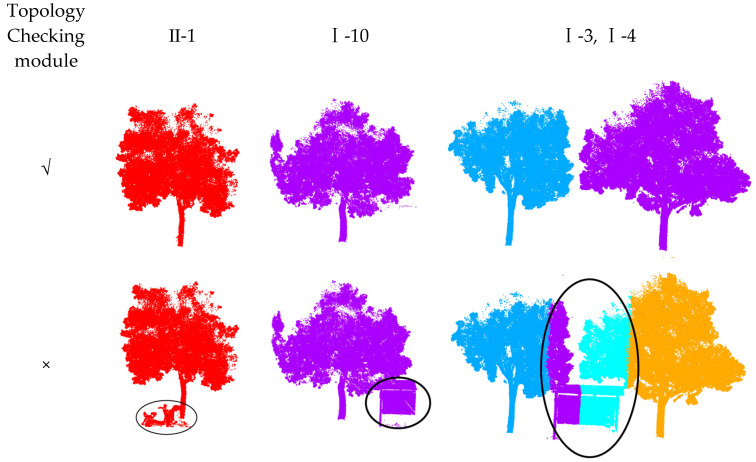
Ablation experiments of the topology checking module. The circle parts are incorrect segmentation.

To further demonstrate the superiority of the proposed algorithm in individual tree extraction, this paper compares and tests the individual tree segmentation algorithm proposed by Hui [42] with the proposed algorithm [10,17]. The test results are shown in Table 5, and the comparative effects of algorithms on both sides of the road are shown in Figure 20 and Figure 21, respectively. Although the comparison algorithm can ensure the effective recognition of all trees in the scene, its average accuracy of individual tree extraction is lower than the proposed algorithm. Figure 20, Figure 21 and Figure 22 also indicate that the proposed algorithm outperforms the comparison algorithm regarding extraction performance. The comparison algorithm first uses DBSCAN to cluster tree trunk point clouds to confirm the position of trees in the scene. Secondly, the crown size is adaptively estimated through the trunk and optimized based on hierarchical mean shift. Therefore, in Figure 20f, the comparison algorithm for individual tree extraction results approximates the crown convex hull to a regular geometric circle or a regular geometric ellipse. However, achieving individual tree segmentation through regular crown geometry results in more tree branches and leaves being separated, resulting in generally low *R* values in the extraction results, affecting the final extraction accuracy.

Overall, the algorithm proposed in this paper can precisely segment street trees. At the same time, compared to the comparison algorithm, the proposed algorithm retains relatively complete edge branch and leaf features, which is crucial for accurately measuring tree crown area.

## 6. Discussion

The discussion mainly discusses the effectiveness of the proposed topology checking module. In addition, we also analyze the applicability of the proposed algorithm on the airborne laser scanning (ALS) point clouds data.

### 6.1. The Effectiveness of the Topology Checking Module

Since there may be disturbing features such as vehicles or signs around the trees, it is necessary to design relevant modules to analyze the initial extraction results and eliminate the interferences. This section verifies the effectiveness of the topology checking module by comparing the processing effect of the module with or without topology.

As shown in the Figure 22, in the absence of the topology checking module in dataset 2 II-1 and I-10, the electric vehicles or signs next to the tree trunks will be misjudged as tree point clouds, and the longer signs in I-3 and I-4 will affect the subsequent limit analysis and processing process, so that the signs will be misjudged as tree point clouds. In summary, the topology checking module can effectively inspect the disturbing features such as electric vehicles and signs next to the analysis tree and remove the interference through voxelization.

### 6.2. The Applicability on ALS Data

**Figure 23:**
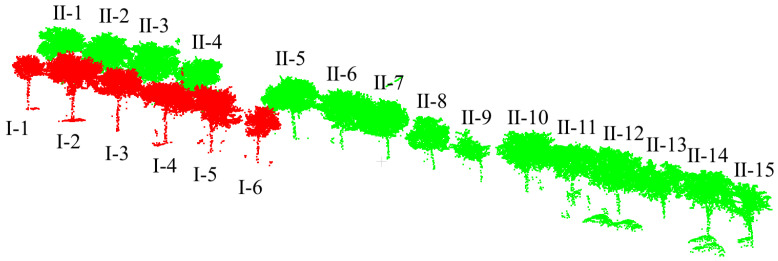
Dublin dataset of ALS data.

The Dublin dataset contains data from ALS acquired via helicopter aerial photography in 2015, focusing on a densely populated area of approximately 2 square kilometers in the center of Dublin. The dataset includes an initial LiDAR point cloud of over 1.4 billion points, which is split into smaller pieces for easier management and processing. The average density of the point cloud is between 250 and 348 points/m^2^. In addition, more than 260 million laser scan points in the dataset are manually annotated into about 100,000 objects, which are classified into 13 categories: buildings, trees, facades, windows, and streets. The vegetation class includes all types of separable plants, including grass, bushes, and trees. As shown in Figure 23, part of the Dublin sample data contains 21 street trees and 197,210 point clouds.

The Dublin street tree plot test results show that the algorithm in this paper can also accurately extract all street trees in the test scene, and the extraction effect is better with an average extraction accuracy of 99.38%. In Figure 23, Table 6 and Table 7, the recall of I-2 is relatively low because the edge branches and leaves are divided into I-3 in I-2. At the same time, the fineness of the boundary division of II-6, II-7, II-10, II-11, and II-12 trees is not high, resulting in some branches and leaf point clouds of II-10 and II-11 being divided into II-11 and II-12, respectively. There are 21 street trees in Dublin. Among them, 14 trees are completely correctly predicted. The remaining seven trees have over-segmentation to varying degrees. In addition, all trees have under-segmentation. It can be seen that under-segmentation is still more common in the mis-segmentation operation in the Dublin street tree plot data.

## 7. Conclusions

This article uses LiDAR-scanned street tree point cloud data to complete the individual tree segmentation and parameter extraction of street trees. Firstly, the initial segmentation of the tree scene was constructed using the DBSCAN algorithm. Then, we optimized the initial segmentation results and detected their under-segmentation. Finally, limit detection on under-segmented trees is performed to segment street trees effectively. In addition, this article designs a series of individual tree parameter extraction algorithms to effectively extract the height, DBH, and crown area of an individual tree, completing the automated processing from obtaining street tree point clouds to obtaining street tree individual tree parameters, which is helpful for inexperienced staff to implement.

## Figures and Tables

**Table 1:** Quantitative evaluation of the precision of individual tree segmentation test results of dataset 1.

	Predict	*P* (%)	*R* (%)	*F*_1_ (%)
Type				
I-1		100.00	97.15	98.55
I-2		100.00	97.38	98.67
I-3		100.00	95.12	97.50
I-4		98.77	99.59	99.18
I-5		98.67	99.87	99.27
I-6		99.33	98.76	99.04
I-7		99.64	98.74	99.19
I-8		99.85	99.11	99.48
I-9		100.00	97.57	98.77
I-10		100.00	99.46	99.73
I-11		99.98	98.22	99.09
I-12		99.76	98.92	99.34
II-1		99.87	98.39	99.12
II-2		99.98	99.52	99.75
II-3		100.00	99.69	99.84
II-4		99.82	98.99	99.40
II-5		89.41	99.49	94.18
II-6		99.73	99.97	99.85
II-7		99.79	99.72	99.75
II-8		98.95	99.55	99.25
II-9		100.00	99.80	99.90
II-10		100.00	100.00	100.00
II-11		99.34	99.96	99.65
II-12		100.00	99.30	99.65
II-13		100.00	97.83	98.90
II-14		97.77	99.97	98.86
Average		99.26	98.93	99.07

**Table 2:** Individual tree parameter extraction results of dataset 1.

Index	Tree Height (m) /AIoU	DBH (m) /AIoU	Tree Radius (m) /AIoU	Crown Area (m^2^) /AIoU
I-1	12.77/100%	0.25/100%	0.13/100%	20.52/100%
I-2	7.71/100%	0.21/100%	0.11/100%	14.27/100%
I-3	12.14/100%	0.26/100%	0.13/100%	31.89/93.43%
I-4	10.33/100%	0.21/100%	0.11/100%	24.93/94.12%
I-5	11.85/100%	0.24/100%	0.12/100%	35.92/98.58%
I-6	7.88/100%	0.16/100%	0.08/100%	18.23/97.63%
I-7	9.08/100%	0.17/100%	0.09/100%	25.41/100%
I-8	11.92/100%	0.25/100%	0.12/100%	37.26/95.54%
I-9	9.44/100%	0.15/100%	0.08/100%	15.84/100%
I-10	11.24/100%	0.15/100%	0.08/100%	14.88/99.25%
I-11	11.40/100%	0.23/100%	0.11/100%	30.94/97.37%
I-12	9.27/100%	0.17/100%	0.09/100%	22.25/95.36%
II-1	8.98/100%	0.17/100%	0.08/100%	22.50/100%
II-2	8.36/100%	2.21/100%	1.10/100%	23.88/100%
II-3	6.18/100%	0.14/100%	0.07/100%	9.63/100%
II-4	12.35/100%	0.26/100%	0.13/100%	35.55/100%
II-5	8.01/100%	0.14/100%	0.07/100%	13.55/94.54%
II-6	9.24/100%	0.12/100%	0.06/100%	21.47/100%
II-7	14.51/100%	0.22/100%	0.11/100%	38.29/100%
II-8	11.27/100%	0.93/100%	0.47/100%	18.90/100%
II-9	9.27/100%	1.66/100%	0.83/100%	15.91/100%
II-10	6.52/100%	1.41/100%	0.71/100%	10.32/100%
II-11	9.07/100%	0.16/100%	0.08/100%	12.88/100%
II-12	7.68/100%	0.14/100%	0.07/100%	13.00/100%
II-13	7.45/100%	0.21/100%	0.10/100%	16.61/100%
II-14	12.69/100%	0.22/100%	0.11/100%	49.00/100%

**Table 3:** Quantitative evaluation of individual tree segmentation test results of dataset 2.

	Prediction	*P* (%)	*R* (%)	*F*_1_ (%)
Type				
I-1		99.99	99.96	99.98
I-2		99.99	99.99	99.99
I-3		99.99	98.07	99.02
I-4		99.99	98.82	99.40
I-5		100.00	99.83	99.91
I-6		99.95	99.99	99.97
I-7		99.80	99.92	99.86
I-8		99.99	99.99	99.99
I-9		99.99	100.00	99.99
I-10		99.65	99.99	99.82
I-11		98.74	98.64	98.69
I-12		98.87	99.98	99.42
II-1		99.99	99.93	99.96
II-2		100.00	98.64	99.31
II-3		98.83	99.99	99.41
II-4		99.41	99.31	99.36
II-5		96.71	98.20	97.45
II-6		100.00	96.16	98.04
II-7		99.99	100.00	99.99
II-8		99.99	99.99	99.99
II-9		99.99	99.95	99.97
II-10		99.99	100.00	99.99
II-11		99.99	100.00	99.99
II-12		100.00	100.00	100.00
II-13		99.91	99.71	99.81
II-14		99.99	99.93	99.96
Average		99.99	99.21	99.66

**Table 4:** Individual tree parameter extraction results of dataset 2.

Index	Tree Height (m) /AIoU	DBH (m) /AIoU	Tree Radius (m) /AIoU	Crown Area (m^2^) /AIoU
I-1	6.25/100%	0.20/100%	0.10/100%	22.45/100%
I-2	7.36/100%	0.26/100%	0.13/100%	20.78/100%
I-3	7.16/100%	0.26/100%	0.13/100%	24.80/100%
I-4	7.80/100%	0.32/100%	0.16/100%	33.53/100%
I-5	7.45/100%	0.26/100%	0.13/100%	19.35/100%
I-6	9.07/100%	0.3/100%	0.15/100%	33.42/100%
I-7	6.97/100%	0.08/100%	0.04/100%	23.00/100%
I-8	6.84/100%	0.08/100%	0.04/100%	21.67/100%
I-9	7.88/100%	0.06/100%	0.03/100%	16.98/91%
I-10	6.63/100%	0.08/100%	0.04/100%	26.49/90%
II-1	8.16/100%	0.06/100%	0.03/100%	29.76/100%
II-2	7.98/100%	0.08/100%	0.04/100%	37.99/100%
II-3	6.68/100%	0.08/100%	0.04/100%	27.90/98%
II-4	6.35/100%	0.06/100%	0.03/100%	17.09/98%
II-5	7.75/100%	0.06/100%	0.03/100%	28.42/100%
II-6	7.14/100%	0.08/100%	0.04/100%	32.76/100%
II-7	7.28/100%	0.28/100%	0.14/100%	36.75/100%
II-8	7.65/100%	0.10/100%	0.05/100%	26.29/100%
II-9	5.44/100%	0.06/100%	0.03/100%	13.95/100%
II-10	7.78/100%	0.10/100%	0.05/100%	44.10/100%
II-11	5.95/100%	0.06/100%	0.03/100%	13.41/100%
II-12	5.86/100%	0.06/100%	0.03/100%	16.67/100%
II-13	8.13/100%	0.10/100%	0.05/100%	35.46/100%
II-14	5.86/100%	0.08/100%	0.04/100%	20.25/96%
II-15	6.03/100%	0.06/100%	0.03/100%	21.80/95%
II-16	6.95/100%	0.06/100%	0.03/100%	32.55/100%

**Table 5:** Comparison results of individual tree segmentation.

	Predict	*P* (%)		*R* (%)		*F*_1_ (%)	
Type		Ours	Comparison Algorithm	Ours	Comparison Algorithm	Ours	Comparison Algorithm
I-1		100.00	99.77	97.15	96.96	98.55	98.35
I-2		100.00	99.02	97.38	57.59	98.67	72.83
I-3		100.00	99.79	95.12	91.88	97.50	95.67
I-4		98.77	99.73	99.59	98.30	99.18	99.01
I-5		98.67	100.00	99.87	98.51	99.27	99.25
I-6		99.33	99.50	98.76	95.91	99.04	97.67
I-7		99.64	100.00	98.74	87.81	99.19	93.51
I-8		99.85	100.00	99.11	98.25	99.48	99.12
I-9		100.00	100.00	97.57	96.14	98.77	98.03
I-10		100.00	99.97	99.46	99.49	99.73	99.73
I-11		99.98	100.00	98.22	96.01	99.09	97.96
I-12		99.76	100.00	98.92	95.15	99.34	97.51
II-1		99.87	99.98	98.39	96.96	99.12	98.45
II-2		99.98	99.39	99.52	95.44	99.75	97.37
II-3		100.00	98.73	99.69	92.86	99.84	95.71
II-4		99.82	97.06	98.99	93.14	99.40	95.06
II-5		89.41	97.08	99.49	90.97	94.18	93.93
II-6		99.73	99.34	99.97	84.05	99.85	91.06
II-7		99.79	99.82	99.72	96.71	99.75	98.24
II-8		98.95	99.19	99.55	95.63	99.25	97.38
II-9		100.00	99.75	99.80	94.33	99.90	96.96
II-10		100.00	99.73	100.00	92.87	100.00	96.18
II-11		99.34	99.8	99.96	90.95	99.65	95.17
II-12		100.00	99.65	99.30	92.61	99.65	96.00
II-13		100.00	99.41	97.83	92.69	98.90	95.93
II-14		97.77	98.60	99.97	95.83	98.86	97.20
Avgerage		99.26	99.44	98.93	92.96	99.07	95.90

**Table 6:** Quantitative evaluation of individual tree segmentation test results of ALS Data.

	Predict	*P* (%)	*R* (%)	*F*_1_ (%)
Type				
I-1		100.00	98.15	99.07
I-2		100.00	97.12	98.54
I-3		100.00	98.42	99.20
I-4		99.87	98.46	99.16
I-5		99.78	99.87	99.82
I-6		100.00	99.76	99.88
II-1		100.00	99.59	99.79
II-2		100.00	99.60	99.80
II-3		100.00	99.88	99.94
II-4		100.00	98.33	99.47
II-5		100.00	99.49	98.16
II-6		99.84	98.54	96.85
II-7		99.79	98.65	99.19
II-8		100.00	99.56	99.78
II-9		100.00	99.90	99.95
II-10		99.80	99.75	99.77
II-11		99.69	99.89	99.85
II-12		99.88	99.56	99.79
II-13		100.00	99.63	99.81
II-14		100.00	98.69	99.34
II-15		100.00	99.59	99.79
Average		99.94	99.16	99.38

**Table 7:** Individual tree parameter extraction results of ALS data.

Index	Tree Height (m)	DBH (m)	Tree Radius (m)	Crown Area (m^2^)
I-1	5.74	0.21	0.11	7.98
I-2	7.05	0.21	0.10	23.91
I-3	6.51	0.25	0.12	14.50
I-4	6.42	0.16	0.08	18.58
I-5	6.94	0.23	0.12	19.98
I-6	5.96	0.22	0.11	10.58
II-1	5.69	0.21	0.11	16.56
II-2	5.87	0.12	0.06	21.06
II-3	6.26	0.29	0.15	22.69
II-4	5.60	0.17	0.08	17.02
II-5	6.19	0.19	0.10	23.84
II-6	5.97	0.11	0.06	23.41
II-7	5.80	0.34	0.17	25.03
II-8	5.15	0.18	0.09	17.16
II-9	5.15	0.16	0.08	9.70
II-10	6.32	0.64	0.32	24.94
II-11	6.09	0.27	0.14	24.48
II-12	6.55	0.20	0.10	27.11
II-13	5.66	0.30	0.15	22.72
II-14	6.58	0.34	0.17	23.33
II-15	6.38	0.57	0.28	15.95

## Data Availability

Data are contained within the article.
